# The Microbial Profile of Keloid Tissue: A Potential Biomarker for Lesion Activity

**DOI:** 10.3390/biomedicines14020386

**Published:** 2026-02-07

**Authors:** Wenjie Xia, Sihui Wang, Yang Xu, Hui Hua, Rong Guo, Bingrong Zhou

**Affiliations:** 1 Department of Dermatology, The First Affiliated Hospital with Nanjing Medical University, Nanjing 210029, China; 19180126@stu.njmu.edu.cn (W.X.); wsh1121@stu.njmu.edu.cn (S.W.); xuyang@stu.njmu.edu.cn (Y.X.); 2 Department of Dermatology, Nantong Third People’s Hospital, Affiliated Nantong Hospital 3 of Nantong University, Nantong 226006, China; huahui@ntu.edu.cn; 3 Department of Dermatology, Shanxi Bethune Hospital, Shanxi Academy of Medical Sciences, Third Hospital of Shanxi Medical University, Tongji Shanxi Hospital, Taiyuan 030032, China

**Keywords:** keloid, disease activity, microbiota, 16S rRNA sequencing

## Abstract

**Background**: Keloids can extend beyond the boundaries of the original wound and often cause itching or pain. Although the skin microbiome is known to influence various skin conditions, it is still unclear how the microbiota inside keloid tissues differ between active and inactive stages. **Methods**: We enrolled 43 patients with active keloids and 20 patients with inactive lesions. Tissue samples were collected from keloids and from nearby normal skin. In active lesions, both the relatively unstable and stable regions were also sampled. Microbial composition and predicted functions were analyzed using 16S Ribosomal RNA (rRNA) sequencing and standard bioinformatic approaches. **Results**: Active keloids exhibited a distinct microbial profile compared to normal skin. *Acinetobacter* and *Pseudomonas* were more abundant in active lesions, while *Cutibacterium* was more common in normal skin. Functional prediction also indicated changes in lipid-related pathways in active lesions. In contrast, inactive keloids showed no significant differences from normal skin, and different regions within active lesions had similar microbial features. **Conclusions**: This study indicates that alterations in the microbiota are linked to the activity of keloids. Potential microbiome-based translational pathways should be explored for monitoring and managing keloid activity in the future.

## 1. Introduction

Human skin hosts a complex ecosystem with commensal microbes, whose homeostasis promotes skin barrier integrity and wound repair through multi-dimensional mechanisms, such as competitive colonization, secretion of antibacterial substances and regulation of immune cell function [1,2]. The composition of the skin microbiome is affected by multiple internal and external factors, including age, gender, genetics, environmental exposure, lifestyle, and antibiotic use [2]. A breakdown in this balance compromises the skin barrier and derails immune responses, resulting in persistent inflammation and delayed healing [3].

Keloids are pathological scars that arise after skin injury or, in some cases, without a clear trigger. They grow beyond the original wound and are marked by abnormal proliferation of fibroblasts accompanied by excessive deposition of extracellular matrix. Clinically, they often extend progressively and cause itching or pain, with a high likelihood of recurrence, substantially impacting patients’ life quality [4]. Although genetic predisposition, overactivation of Transforming Growth Factor-β (TGF-β)/Smad signaling, and local immune imbalance are known to contribute to keloid formation, the factors that initiate and sustain these changes remain unclear [4]. Based on their clinical behavior, keloids have been categorized as active or inactive. Active lesions typically show ongoing enlargement with symptoms such as itching and pain, while inactive lesions remain relatively stable and asymptomatic [5]. Even within the same keloid, regional differences exist: the peripheral zone often appears red and raised with active growth, whereas the central area tends to be flatter and less symptomatic [6]. These patterns suggest that different local microenvironments may shape keloid behavior.

Several studies have begun to explore the microbial features of keloids. Zhang et al. [7] reported that keloid tissue harbors distinct bacterial colonization patterns and suggested that microbial imbalance might promote fibroblast migration, collagen deposition, and tissue contraction through the interleukin-8 (IL-8)/CXCR1/2 pathway. Chen et al. [8] found that infected keloids contain different dominant bacterial groups on the surface compared with deeper tissue, with Actinobacteria, Propionibacteriales, and Corynebacteriales enriched on the surface, and *Staphylococcus*, *Peptoniphilus*, and *Cutibacterium* enriched within lesions, potentially contributing to inflammation and tumor-like growth. Shan et al. [9] identified an enrichment of catalase (CAT)-negative bacteria in keloids and showed that reduced CAT activity may promote fibroblast proliferation and migration. However, existing studies have relied on mixed samples without stratifying for disease activity. It therefore remains unclear whether microbial changes are linked to lesion activity or how microbiota are distributed across different regions within the same lesion.

To address these gaps, we systematically compared the microbiota structure, identified potential biomarkers and predicted functional pathways of keloids with different activity. Our goal was to identify potential biomarkers related to keloid activity and provide a foundation for future mechanistic studies and intervention research.

## 2. Materials and Methods

### 2.1. Patient Cohort and Definition of Keloid Activity

This study was approved by the Ethics Committee of Shanxi Taiyuan Vitiligo Hospital (Approval No. 2025-01). All enrolled patients had a clinical and pathological diagnosis of keloid, aged 18 to 60 years, and signed informed consent. Individuals who had used antibiotics or immunosuppressants within the past 3 months or had systemic or infectious skin diseases were excluded. Clinical data collection included age, gender, body mass index (BMI), disease course, location, diameter, and numerical rating scale (NRS) score of pain or itching through medical interviews. The NRS quantifies current pain intensity on a scale from 0 to 10, with a score of ≤3 considered controlled pain [10,11]. Meanwhile, the Vancouver Scar Scale (VSS) was used to assess the severity of keloid [12].

**Figure 1:**
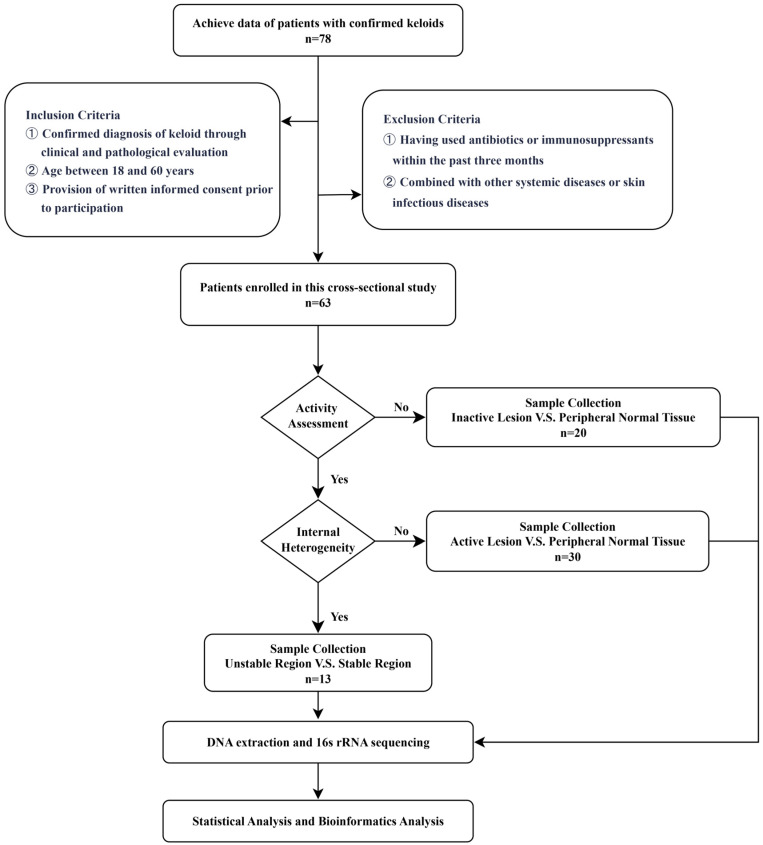
Flow chart of keloid sample collection and data analysis. A total of 63 patients were enrolled, from whom 43 active keloids and 20 inactive keloids were collected and classified. The active cases were sampled using two strategies: in 30 cases, paired samples were collected from the lesion and the peripheral normal skin, while in another 13 cases, paired samples were taken from relatively stable and unstable areas within the lesion. Inactive cases provided 20 pairs of lesion and peripheral normal skin samples.

Referring to previously described criteria [5] and our clinical observations, we defined the activity of keloid as continuous or intermittent growth, pain, and itching (NRS score ≥ 3) of the lesion in the past 6 months. Inactivity was defined as a stable lesion size and no significant subjective symptoms for at least 6 months. A flow chart of the overall procedure is presented in Figure 1.

### 2.2. Sample Collection and Processing

Under strict aseptic conditions during surgery, approximately 0.5 cm^3^ full-thickness skin tissue samples were excised after iodophor disinfection. These tissues were immediately flash-frozen in liquid nitrogen and then transferred to −80 °C refrigerator for storage until DNA was extracted. Samples were obtained from three groups: the first consisted of active keloid tissue and adjacent normal skin, which was sampled from an area more than 0.5 cm away from the keloid margin to ensure it was unaffected. The second group included paired tissues from the relatively unstable and stable areas within an active keloid. The third group comprised inactive keloid tissue and its corresponding normal skin, also obtained from a site over 0.5 cm beyond the edge of the lesion. All procedures were performed carefully to minimize contamination and preserve microbial integrity.

### 2.3. DNA Extraction and 16S rRNA Amplicon Sequencing

#### 2.3.1. Genomic DNA Extraction

Genomic DNA was extracted using the CTAB/SDS method under strict sterile conditions; all solutions and equipment were sterile, the DNA extraction kit was freshly opened, and samples were processed in areas physically separated from the PCR amplification region. Next, the DNA quality was checked on 1% agarose gels, and the samples were then diluted to 1 ng/µL for subsequent analysis.

#### 2.3.2. 16S rRNA Gene Amplification and Sequencing

The 16S rRNA gene was amplified in different regions (16S V34) using specific primers (16S V34:341F, 806R) and barcodes. All PCR mixtures contained 15 μL of Phusion^®^ High-Fidelity PCR Master Mix (New England Biolabs, Ipswich, MA, USA), 0.2 μM of each primer and 10 ng target DNA. The thermal cycling protocol comprised an initial 98 °C for 1 min, 30 cycles of (98 °C for 10 s, 50 °C for 30 s, 72 °C for 30 s), and a final 72 °C for 5 min.

#### 2.3.3. Quantification and Identification of PCR Products

The PCR products were verified by electrophoresis on a 2% agarose gel and then mixed in equal amounts before purification using the Qiagen Gel Extraction Kit (Qiagen, Hilden, Germany).

#### 2.3.4. Library Preparation and Sequencing

Sequencing libraries were constructed using the NEBNext Ultra II kit (Cat No. E7645, New England Biolabs, Ipswich, MA, USA), assessed for quality, and sequenced on an Illumina NovaSeq platform to generate 250 bp paired-end reads.

### 2.4. Sequencing Data Processing

#### 2.4.1. Data Preprocessing

Raw reads were processed using the EasyAmplicon pipeline [13]. The double-end reads were spliced through Vsearch (v2.30.0), and the sequence was renamed according to the sample identification. After primer removal and filtering, high-quality sequences within the expected amplicon region were retained for subsequent analysis.

#### 2.4.2. ASV Generation and Species Annotation

Denoising was performed using the unoise3 algorithm in USEARCH (v12) to generate amplicon sequence variants (ASVs). Low-abundance noise was removed using a minimum count threshold of 10. Taxonomic assignment was performed against the RDP 16S database (v18), and non-bacterial sequences were excluded.

#### 2.4.3. Stacking Map Drawing and Diversity Analysis of Species Composition

Taxonomic annotations were combined with ASV abundance data to calculate relative abundances at various taxonomic levels, and then the ggplot2 R package (v3.5.2) was used to draw a stacked map of species composition. In addition, Simpson’s evenness index was calculated to evaluate the alpha diversity of the microbiota within the sample. Beta diversity was analyzed through principal coordinate analysis (PCoA) using a Bray–Curtis distance matrix, in conjunction with a permuted multivariate analysis of variance (PERMANOVA) to test for significant differences in microbiota structure between groups.

#### 2.4.4. Microbial Association Network Analysis

The CoNet tool (v1.1.1.beta) (multiple measures based on Spearman and Pearson correlation, Bray–Curtis and Kullback–Leibler distance) was used to construct the microbial association network between active keloid and normal skin. Only robust correlations with *p* < 0.05 after correction for multiple testing (Benjamini–Hochberg FDR correction) were retained, and the networks were visualized using Cytoscape (v3.9.1).

#### 2.4.5. Identification of Biomarker

To identify the significant difference between the two groups, the Linear discriminant analysis Effect Size (LEfSe) method was used with an LDA score > 2.0, and the Wilcoxon rank sum test was used to compare the relative abundance of dominant bacteria.

#### 2.4.6. Phylogenetic Tree Construction and Visualization

High-abundance ASVs (>0.2%) were aligned using Muscle software (v5.2), and a maximum likelihood phylogenetic tree was built using IQ-TREE with 1000 bootstrap repeated tests. The tree was finally visualized using the iTOL online platform.

#### 2.4.7. Functional Prediction and Phenotypic Prediction

Based on PICRUSt2 (v2.3.0-b), the pathway_daa function in the ggpicrust2 R package (v1.7.4) [14], combined with the linear model differential abundance analysis (LinDA) method, was used to identify differentially abundant Kyoto Encyclopedia of Genes and Genomes (KEGG) pathways (*p* < 0.05). GreenGenes (v13_8_99) was used to annotate the ASV feature table, and BugBase [15] was then used to classify the microbiota into seven phenotypes.

### 2.5. Statistical Analysis

All statistical analyses were performed with SPSS 26.0 statistical software and R (v4.4.1). The Shapiro–Wilk test was used to evaluate the normality of continuous variables. Non-normally distributed continuous variables were expressed as median (quartile) [*M* (P_25_, P_75_)], and comparisons between two groups were performed using the Mann–Whitney U test. Categorical variables were described by count and constituent ratio (%), and group differences were analyzed using the chi-square test. Grade data, such as symptom scores, were expressed as *M* (P_25_, P_75_), and compared between groups using the Mann–Whitney U test. Statistical significance was considered at *p* < 0.05.

Alpha diversity was assessed by the Simpson evenness index, and groups were compared using ANOVA analysis of variance, with post hoc testing conducted via the Tukey HSD method. Between-group differences in beta diversity were verified by PERMANOVA. Microbial association networks were analyzed using the CoNet tool, integrating Spearman and Pearson correlation coefficients, Bray–Curtis and Kullback–Leibler distance measures, and applying Benjamini–Hochberg FDR correction to control the false discovery rate. The differential flora was identified by the LEfSe method (LDA score > 2.0) and the Wilcoxon rank sum test. For functional prediction, PICRUSt2 and LinDA were used to screen the differentially expressed KEGG pathways between groups. The threshold for statistical significance was set at *p* < 0.05.

## 3. Results

### 3.1. Analysis of Baseline Characteristics

**Figure 2:**
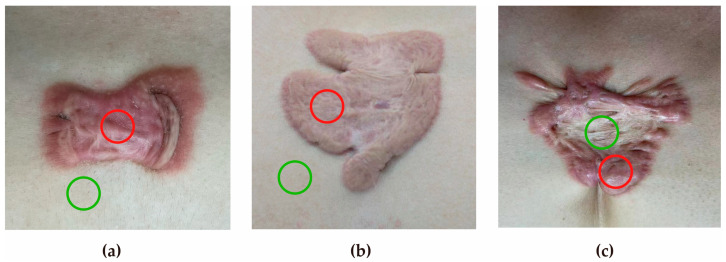
Clinical images. (**a**) Active keloid: lesion and peripheral skin samples. The red circle is the active keloid, and the green circle is the peripheral normal skin. (**b**) Inactive keloid: lesion and peripheral skin samples. The red circle is the inactive keloid, and the green circle is the peripheral normal skin. (**c**) Active keloid: the relatively unstable and stable area inside the lesion, the red circle is the unstable area, and the green circle is the stable area.

Clinical images are provided in Figure 2, and full demographic and clinical data are compiled in Table 1. Compared with the inactive group, the active group showed significant differences in VSS scores, as well as pain and itching NRS scores. Other baseline characteristics, such as age, sex, BMI, disease duration, lesion location and size, were similar between the two groups.

### 3.2. Difference in Microbiota Between Active Keloid and Peripheral Normal Skin

#### 3.2.1. Composition Characteristics of Microbiota

**Figure 3:**
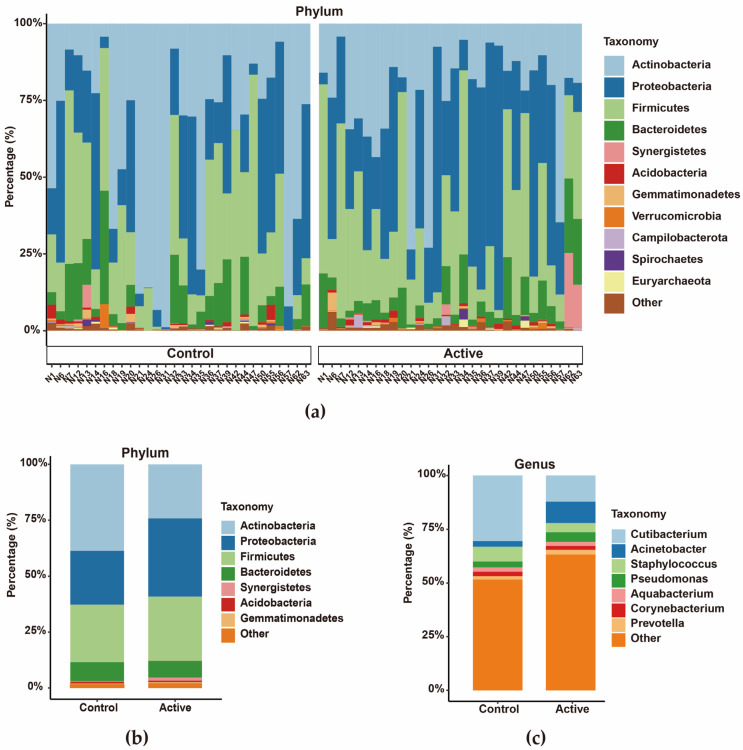
Stacked column bar graph depicting the microbial composition in active keloid and peripheral normal skin (*n* = 30). (**a**) Composition at the phylum level for individual samples. (**b**) Group-averaged composition at the phylum level. (**c**) Composition at the genus level by group.

Active keloids showed a clear shift in microbial composition compared with adjacent normal skin. Proteobacteria and Firmicutes were more abundant in active lesions, whereas Actinobacteria were reduced (Figure 3). In contrast to normal skin, where *Cutibacterium* was the dominant genus, active lesions showed significant enrichment of *Acinetobacter* and *Pseudomonas* (Figure 3c).

#### 3.2.2. Microbiota Diversity Analysis

**Figure 4:**
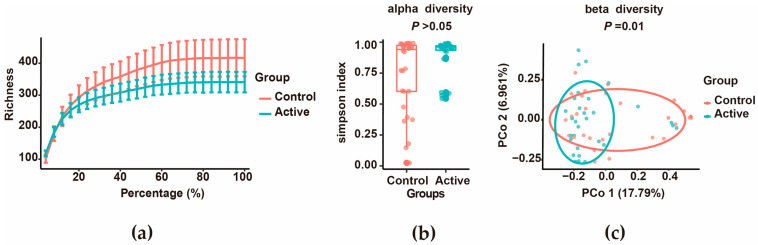
Microbiota diversity analysis. (**a**) Rarefaction curves: With the increase in sequencing volume, the rarefaction curves of both groups tended to be saturated. (**b**) Alpha diversity comparison: Boxplot of Simpson index comparison between active keloid group and peripheral normal skin group (*p* > 0.05, ANOVA followed by Tukey’s HSD correction). The horizontal bar within the box indicates the median. The top and bottom of the box represent the 75th and 25th percentiles, respectively. The upper and lower whiskers extend to data no more than 1.5 times the interquartile range from the upper and lower edges of the box, respectively. The number of samples in the figure represents active (*n* = 30) and control (*n* = 30). (**c**) Beta diversity analysis: PCoA plot based on Bray–Curtis distance (PC1 vs. PC2); PC1 explained 17.79% of the variation, PC2 explained 6.96% of the variation. Ellipse indicates 95% confidence intervals. PERMANOVA test showed significant differences in microbial composition between groups (*p* = 0.01).

Sequencing depth was sufficient for all samples, as indicated by saturation of rarefaction curves (Figure 4a). Alpha diversity did not differ significantly between active lesions and adjacent skin (Figure 4b). However, beta diversity analysis revealed a clear separation between the two groups, and this difference was significant according to PERMANOVA (*p* = 0.01, Figure 4c).

#### 3.2.3. Structure of the Microbial Association Network

**Figure 5:**
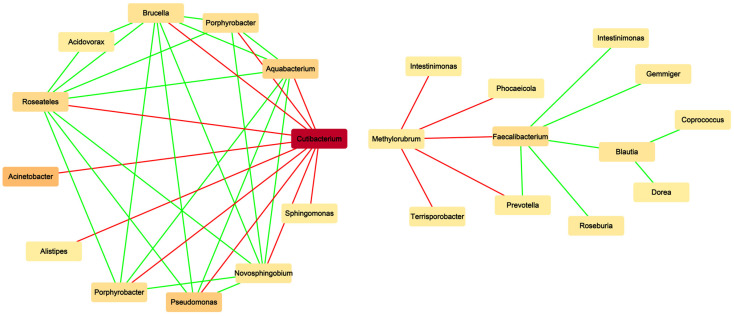
Microbial association network analysis. The red line represents negative correlation and the green line represents positive correlation. Color depth was positively correlated with genus abundance.

The microbial association network of active keloid tissue and peripheral normal skin showed a significant negative relationship between *Cutibacterium* and *Pseudomonas* (*p* < 0.05, Figure 5), suggesting a shift in local microbial interactions.

#### 3.2.4. Biomarker Identification and Phylogenetic Tree Analysis

**Figure 6:**
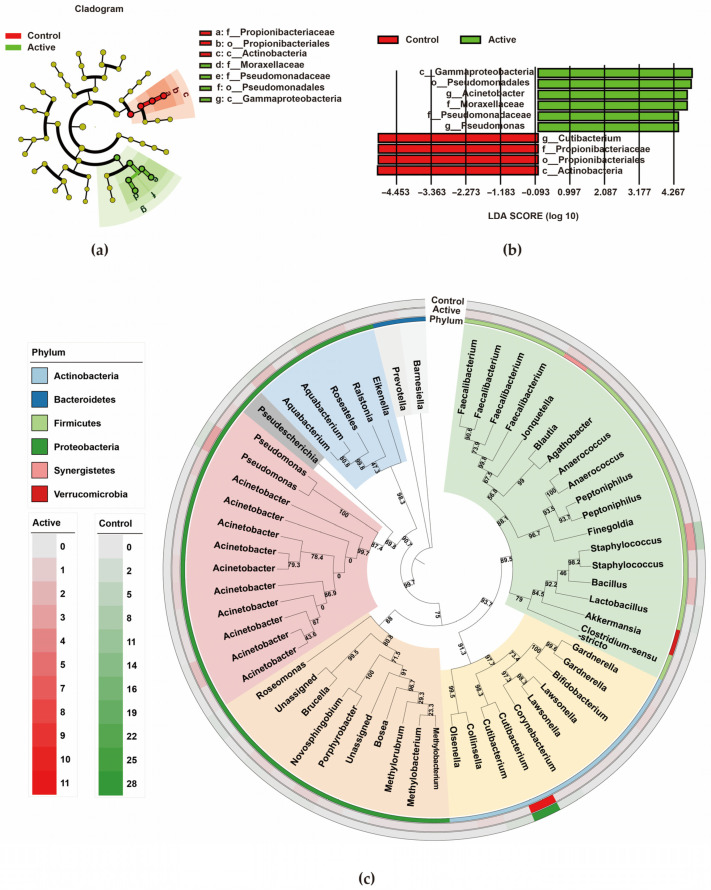
LEfSe analysis and phylogenetic tree construction were performed. (**a**) Evolutionary branch plot. (**b**) Bar plot of LDA value distribution. LEfSe analysis identified species with significant differential abundance between the two groups, and the bar graph length represents the effect size for species with significant differences. (**c**) The phylogenetic tree, constructed from ASV representative sequences, illustrates the genetic relationship between each species. The branch represents the evolutionary relationship composed of two or more organisms, each node represents a taxon, and the corresponding bootstrap is marked to evaluate the credibility of the branch.

Identification of microbial biomarkers via LEfSe analysis (Figure 6a,b) revealed three marker genera with distinct abundances between the groups. Among them, *Acinetobacter* and *Pseudomonas* were significantly enriched in active keloids, while *Cutibacterium* was enriched in the peripheral normal skin. The phylogenetic tree (Figure 6c) showed that *Acinetobacter* and *Pseudomonas* clustered closely, indicating a similar evolutionary background.

#### 3.2.5. Prediction of Function and Phenotype

**Figure 7:**
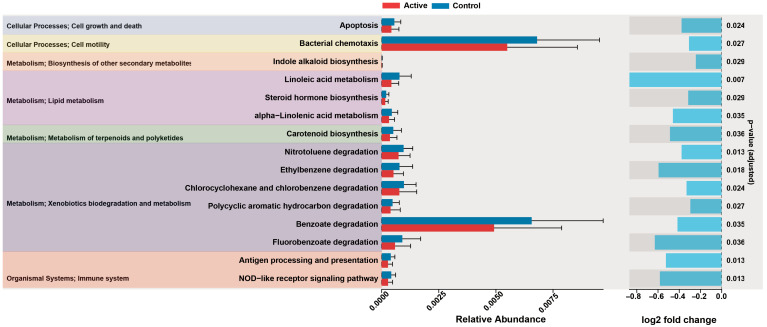
Difference in predicted KEGG pathway abundance between active keloids and peripheral normal skin. Based on 16S rRNA gene sequencing data, metabolic pathways with significant differences in relative abundance between groups were predicted and displayed by PICRUSt2. Error bars indicate the range of variation in pathway abundance. Annotated *p*-values versus log2FC show the significance of the difference derived from LinDA analysis.

**Figure 8:**
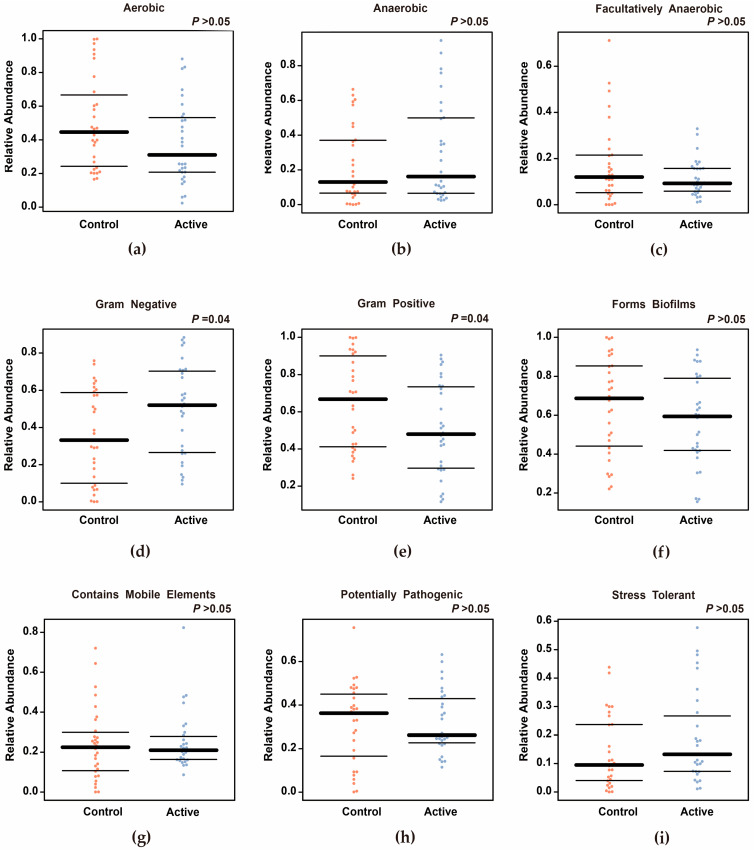
The difference analysis chart of bacterial phenotype prediction based on Bugbase between groups. The functional analysis of microbial communities was conducted using the ASV characteristic table, and the differences in the microbiota in different functional characteristicswere evaluated. The abundance of each phenotype is expressed as the relative abundance of the microbes carrying that trait. (**a**) Aerobic bacteria. (**b**) Anaerobic bacteria (**c**) Facultatively anaerobic bacteria (**d**) Gram-negative bacteria (**e**) Gram-positive bacteria (**f**) Biofilm-forming bacteria (**g**) Bacteria containing mobile genetic elements (**h**) Potentially pathogenic bacteria (**i**) Stress tolerant bacteria. The abundance of Gram-negative bacteria in active keloids increases (*p* = 0.04), while the abundance of Gram-positive bacteria decreases (*p* = 0.04), suggesting changes in the microenvironment.

KEGG pathway prediction analysis based on PICRUSt2 showed that there were significant differences in multiple functional pathways between active keloid and peripheral normal skin (*p* < 0.05, Figure 7). Among them, the abundance of pathways such as lipid metabolism and xenobiotic degradation was significantly up-regulated in active keloids. BugBase-based bacterial phenotype prediction (Figure 8) showed that the relative abundance of Gram-negative bacteria increased (*p* = 0.04) and the relative abundance of Gram-positive bacteria decreased (*p* = 0.04) in active keloids. No significant differences were observed for the other phenotypes between the groups (*p* > 0.05).

### 3.3. Comparison of Microbiota Between Inactive Keloid and Peripheral Normal Skin

**Figure 9:**
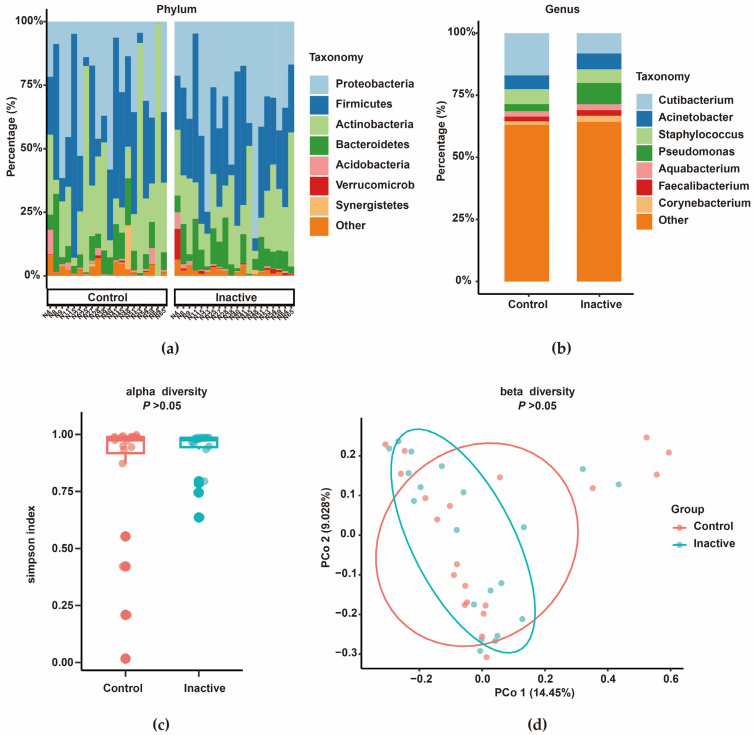
Microbial composition and diversity in inactive keloid and peripheral normal skin (*n* = 20 paired samples). (**a**) Phylum-level abundance in individual samples. (**b**) Genus-level composition by group. (**c**) Alpha diversity (Simpson index) between groups (*p* > 0.05). (**d**) Beta diversity: PCoA plot based on Bray–Curtis distances (PERMANOVA, *p* > 0.05).

There was no significant difference in microbiota between inactive keloids and peripheral normal skin. At the phylum level, Firmicutes, Proteobacteria, and Actinobacteria were the co-dominant phyla in both groups of samples (Figure 9a). At the genus level, the relative abundance of *Pseudomonas* increased in the relatively active region of inactive keloids, whereas the relative abundance of *Cutibacterium* increased in the relatively stable region (Figure 9b). Diversity analysis showed that there was no significant difference in alpha diversity (Figure 9c, Simpson index, *p* > 0.05) and beta diversity (Figure 9d, *p* > 0.05) between the two groups. There was also no significant difference in the abundance of KEGG pathways predicted by PICRUSt2 between the two groups.

### 3.4. Comparison of Microbiota in the Internal Regions of Active Keloids

**Figure 10:**
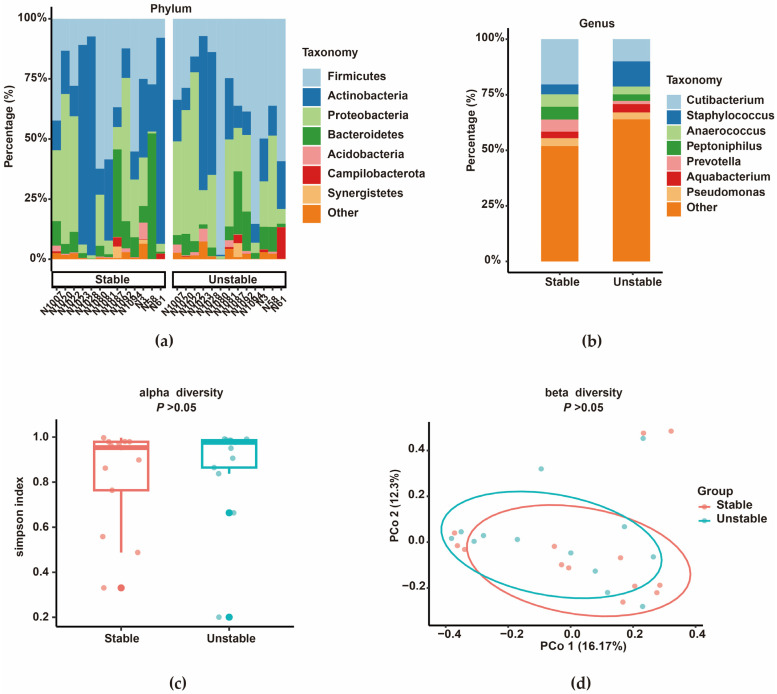
Microbial composition and diversity in relatively unstable and stable regions of active keloids (*n* = 13 paired samples). (**a**) Stacked bar chart showing phylum-level abundance across samples. (**b**) Genus-level composition for each group. (**c**) Alpha diversity, assessed by the Simpson index, showed no significant difference between groups (*p* > 0.05). (**d**) Beta diversity, presented in a PCoA plot (Bray–Curtis distances), also showed no significant separation (PERMANOVA, *p* > 0.05).

The characteristics of the microbiota in the relatively unstable and stable regions of active keloids were highly similar (Figure 10). The microbiota composition at the phylum level was dominated by Proteobacteria, Firmicutes, and Actinobacteria in both groups. (Figure 10a). At the genus level, the relative abundance of *Staphylococcus* increased in the relatively unstable region of active keloids, whereas the relative abundance of *Cutibacterium* increased in the relatively stable region (Figure 10b). Comparative analysis of microbial diversity demonstrated no statistically significant differences in either alpha or beta diversity between the two groups (*p* > 0.05, Figure 10c,d). Functional prediction analysis via PICRUSt2 detected no significant differences in metabolic or enzymatic pathways between the two regions (KEGG pathways, *p* > 0.05).

## 4. Discussion

By stratifying keloids for disease activity, this study identified a potential association between microbiota and lesion activity, an aspect that has not been systematically addressed in previous studies. Unlike previous studies that relied on pooled samples and did not distinguish lesion activity, this study suggests that microbial dysbiosis is not universally present in all keloids but appears to be more closely linked to active lesions. To be specific, the microbiota of active keloids is significantly altered compared to adjacent normal skin, characterized by enrichment of Gram-negative bacteria, such as *Acinetobacter* and *Pseudomonas*, and up-regulation of pathways related to lipid metabolism. These findings are consistent with the possibility of dynamic interactions between microbial composition, immune signaling, and metabolic activity during lesion progression. To sum up, this study modifies the traditional understanding of microbial dysbiosis in keloids, suggesting that it may represent a distinctive trait of active lesions rather than a universal characteristic.

At the genus level, *Acinetobacter* and *Pseudomonas* were significantly enriched in active lesions, while *Cutibacterium* was dominant in normal skin. Gram-negative bacteria such as *Acinetobacter* and *Pseudomonas* usually contain lipopolysaccharide (LPS) components and are often reported to associate with host innate immune pathways such as Toll-like receptor 4(TLR4)-related pathways [16,17,18,19]. *Cutibacterium* is often considered to be related to homeostasis and some anti-inflammatory mechanisms in skin microecology [20,21,22]. The negative association we observed between *Cutibacterium* and *Pseudomonas*, together with an overall increase in Gram-negative bacteria, is indicative of a microenvironment that coincides with a more pro-inflammatory tissue environment during the active phase of keloids.

Our functional predictions also suggested alterations in lipid-related pathways in active lesions. Previous metabolomics studies have shown lipid metabolism disorders in keloids, enrichment of linoleic acid (LA) metabolic pathways, and decreased LA content in keloid tissues [23,24]. These findings are consistent with the possibility of an interaction between the microbiota and the local lipid microenvironment of the lesion. However, functional predictions based on 16S sequencing are indirect. Subsequent work combining metagenomics, lipidomics, and cellular assays will be essential for clarifying the potential causal relationship between changes in microbial function and keloid activity.

Interestingly, inactive keloids did not differ from normal skin in either microbial composition or predicted function. This may reflect the relatively stable biological state of inactive keloids. Although inactive keloids are still characterized by excessive deposition of extracellular matrix, fibrosis by itself does not necessarily imply persistent inflammatory activity. In a variety of fibrotic diseases, mature or stable fibrosis has been shown to persist in the presence of attenuated inflammatory signaling and reduced cell renewal [25,26]. Consistent with this concept, previous studies have shown that the proportion of pro-inflammatory fibroblasts was higher in active keloids than in inactive ones [5]. In inactive lesions, we observed the co-occurrence of established fibrosis, reduced inflammatory activity, and a microbial profile comparable to that of surrounding normal skin. This phenomenon also supports the notion that fibrosis more likely represents a structural end-state of tissue remodeling, whereas inflammatory activity reflects a more dynamic and potentially reversible biological process [25,26].

At the same time, our study did not identify significant microbiota differences among regions within active lesions. This null finding could be due to subtle microenvironmental gradients, the resolution limits of current techniques, or inadequate statistical power. In contrast, histological and mechanical studies have shown that keloid marginal regions often show higher cell activity and stress concentration [6,27,28], suggesting that mechanobiological pathways such as integrin/FAK and YAP/TAZ may play a more direct role in driving local fibrosis [29,30,31]. Previous studies have shown that CCN family members (CCN1-5), a group of extracellular matrix-related proteins involved in wound repair and fibrosis, are significantly upregulated in keloids and strongly induced by mechanical stretch [5]. Future studies combining spatial sequencing with in situ mechanical measurements may help unravel these regional differences.

This study provides evidence linking microbial alterations with keloid activity and identifies potential microbial markers of active disease. However, several limitations should be noted. First, 16S rRNA sequencing does not provide species- or strain-level resolution, and PICRUSt2 prediction based on this only reflects the functional potential and cannot directly represent the actual gene expression level or metabolic flux. The results are also limited by the reference genome coverage and species resolution of the 16S sequence. Therefore, the functional pathway findings should be regarded as hypothesis-generating [32]. Second, we did not include specialized negative controls and statistical decontamination [33]. Although tissue samples were collected under sterile conditions and subjected to strict bioinformatics filtering, the potential effects of contamination and individual factors such as hygiene habits, cosmetic use, and environmental exposure on microbial variability cannot be entirely excluded. Moreover, the study population was drawn from a single geographical region, which may limit broader generalizability. Future studies will include samples from multiple centers and apply experimental negative controls and statistical decontamination methods to further enhance contamination control. Third, this study was a cross-sectional design with all samples from the same surgical time point, making it difficult to assess microbial trends over time or make causal inferences. In addition, the clinical “activity” partly depends on the symptoms reported by the patients, which may be biased. If objective imaging tools such as multimodal photosonic-ultrasound imaging systems are used, quantitative parameters related to the activity of keloids are expected to be obtained [34]. Fourth, although we used a combination of sampling strategies, the possible heterogeneity within lesions and the limited sample size and statistical power to detect subtle spatial differences require caution in interpreting the results of within-lesion comparisons. Longitudinal studies incorporating serial sampling and objective activity measures will be essential to further elucidate the relationship between microbial alterations and disease activity.

Taken together, preliminary correlational findings suggest that potential translational pathways for keloid treatment may be explored in the future. If validated, its ultimate goal may be to shift clinical strategies from traditional symptom control to precise regulation based on skin microecology. Realizing this vision will depend on fundamental advances in robust biomarker identification, targeted microbial interventions, and integration of multiomics. Some hypotheses could be explored in future studies. For example, silver-containing dressings may be used to target *Acinetobacter* or *Pseudomonas* [35], to explore personalized treatment based on drill biopsy results, and to competitively inhibit pathogenic bacteria via colonization with the probiotic *Cutibacterium* [36]. On the other hand, the biofilm formation properties of *Pseudomonas* and *Acinetobacter* provide another approach for future mechanistic studies and clinical translation [37]. The feasibility and efficacy of all the above require further validation in subsequent research.

## 5. Conclusions

In summary, our findings suggest that the microbiota characteristics of keloids were potentially correlated with lesion activity, and active lesions showed characteristic signals of *Acinetobacter* and *Pseudomonas* enrichment and functions related to lipid metabolism. Our findings on microbial signatures offer a foundation for future work to understand the interplay between microbes, immunity, and tissue mechanics in keloid progression. With further validation, these insights may inform the development of microbiome-based tools to assess disease activity and guide microecological therapy.

## Figures and Tables

**Table 1:** Characteristics of the participants.

Characteristic	Active Keloid (*n* = 43)	Inactive Keloid (*n* = 20)	*p*-Value
Demographics			
Age [years, *M* (P_25_, P_75_)]	32 (25, 60)	33 (22.25, 50.25)	0.274
BMI [kg/m^2^, *M* (P_25_, P_75_)]	23.99 (21.46, 25.68)	23.50(21.46, 25.68)	0.707
Gender [*n* (%)]			
Male	25 (58.1)	12 (60)	0.889
Female	18 (41.9)	8 (40)	
Keloid Characteristics			
Course [years, *M* (P_25_, P_75_)]	8 (4, 20)	6.5 (5, 8.75)	0.683
Diameter [cm, *M* (P_25_, P_75_)]	3.2 (2, 10.5)	4.1 (2.2, 5.425)	0.824
Severity [VSS score, *M* (P_25_, P_75_)]	7 (7, 8)	5.5 (5, 7)	<0.001
Location [*n* (%)]			
Front chest	29 (67.4)	9 (45)	0.448
Shoulder	5 (11.6)	4 (20)	
Ear	2 (4.7)	0 (0)	
Others	7 (16.3)	7 (35)	
Activity Assessment			
Growth [*n* (%)]	43 (100)	0 (0)	<0.001
Pain [NRS score, *M* (P_25_, P_75_)]	4 (3, 5)	2 (1, 2)	<0.001
Itching [NRS score, *M* (P_25_, P_75_)]	7 (6, 8)	2 (1, 2)	<0.001
Sampling Information			
Lesion-normal skin paired samples [*n* (%)]	30 (69.8)	20 (100)	
Unstable-stable paired samples in the lesion [*n* (%)]	13 (30.2)	0 (0)	

Abbreviations: BMI, body mass index; VSS, Vancouver Scar Scale; NRS, numerical rating scale.

## Data Availability

The original data presented in the study are openly available in [NGDC] at [https://ngdc.cncb.ac.cn/gsa-human/s/kG792Emq, accessed on 4 February 2026] [HRA015166].

## Abbreviations

The following abbreviations are used in this manuscript:

rRNA	Ribosomal RNA
TGF-β	Transforming Growth Factor-β
IL-8	Interleukin-8
CAT	Catalase
KEGG	Kyoto Encyclopedia of Genes and Genomes
BMI	Body Mass Index
NRS	Numerical Rating Scale
VSS	Vancouver Scar Scale
ASV	Amplified Sequence Variants
PCoA	Principal Coordinate Analysis
PERMANOVA	Permuted-Multivariate Analysis of Variance
LEfSe	Linear Discriminant Analysis Effect Size
LinDA	Linear Model Differential Abundance Analysis
LPS	Lipopolysaccharide
TLR-4	Toll-like receptor 4
LA	Linoleic Acid
